# The Mental Well-Being of Graduate Students in Canada: A Scoping Review

**DOI:** 10.1177/08901171251326308

**Published:** 2025-03-12

**Authors:** Elham Javadizadeh, Abram Oudshoorn, Lori Letts, Skye Barbic, Carrie Anne Marshall

**Affiliations:** 1 Social Justice in Mental Health Research Lab, School of Occupational Therapy, Western University, London, ON, Canada; 2 Arthur Labatt School of Nursing, Faculty of Health Science, Western University, London, ON, Canada; 3 School of Rehabilitation Science, Faculty of Health Sciences, McMaster University, Hamilton, ON, Canada; 4 Department of Occupational Science and Occupational Therapy, University of British Columbia, Vancouver, Canada

**Keywords:** mental well-being, graduate students, canada campuses, health policy < opportunity < strategies, scoping review

## Abstract

**Objective:** To review the literature exploring the mental health of graduate students in Canada. Data Source: Articles identified in EMBASE, CINAHL, PsycInfo, Medline, Sociological Abstracts, Nursing and Allied Health, and ERIC.

Study Inclusion and Exclusion Criteria:Two independent reviewers screened articles that: (1) focused on graduate students’ mental wellbeing; (2) used empirical study designs (3) were published in English; (4) were conducted in Canada.

**Data Extraction:** Twenty-two articles met the inclusion criteria. Data was extracted on the following variables: author(s); year of publication; study design; methodology; clinical characteristics of participants; number of participants; demographic characteristics of participants, journal discipline and university.

**Data Synthesis:** We used Dedoose, a qualitative data management program, to perform qualitative content analysis and characterize the data and identify emerging themes.

**Results:** The content analysis led to three related themes from the included studies: Determinants of mental health in graduate students are myriad; 2) Coping Strategies for Graduate Student Stress; and 3) Bridging Support: Enhancing Mental Well-Being.

**Conclusion:** Our study's findings highlight the significance of investigating the mental well-being of graduate students in Canada. This review showed that by promoting mental well-being, universities and institutions can create a supportive atmosphere that encourages open dialogue, provides access to counseling and other mental health resources, and implements strategies to mitigate the challenges faced by graduate students.

## Introduction

Research conducted on graduate students across the globe shows similar high rates of emotional distress.^1,2^ Graduate students are required to manage many responsibilities, such as coursework, research projects, practicum placements, teaching assistantships, and funding applications.^3^ Thus, graduate students commonly face increased stress regarding their academic performance, and this can be further intensified by the specific academic atmosphere they find themselves in.^4^ Beyond any genetic or life history predisposition, graduate students may encounter unique challenges that arise from conducting research and teaching, publishing their work, and searching for employment.^5^ Doctoral graduate students have been noted to face an increased risk of developing mental health disorders, such as depression, compared to similarly educated individuals who are not pursuing graduate studies.^6^ Financial stress is also a prevalent and impactful problem among graduate students, significantly affecting their mental health.^7^ The burden of financial worries can create distractions that hinder their ability to fully engage in coursework, research, and professional development.^7^

Mental health challenges are a common experience for graduate students as demonstrated by a study conducted in the United States, where researchers point out graduate students on average experience a decline in their mental health during their studies.^8^ Another study revealed that more than one-third of the students seek professional help for anxiety or depression issues during their time in graduate school.^9^ This trend requires attention to improve the overall mental well-being of graduate students. The rates of self-reported depression and anxiety are 6 times greater among graduate students than the general population^10^ and higher than their college educated peers of the same age.^4^ A report on the status of graduate student mental health in the Department of Medical Biophysics at University of Toronto found that almost 20% of graduate students reported experiencing suicidal thoughts during their graduate studies. Additionally, 1.5% reported having a suicide plan at least once but didn’t intend to act on it, while 6% stated that they had a suicide plan and intended to die, but ultimately did not follow through with it.^11^

In a national sample of Canadian graduate students, approximately 1 third of students indicated the presence of significant depression symptoms.^12^ This trend requires attention to improve the overall mental wellbeing of graduate students. Mental health problems can adversely affect student outcomes in the short and long term, such as limiting social relationships, reducing academic achievement and retention, and lessening future economic productivity.^13^ Analysis of baseline demographic data from the Ph. D students in US and Canada showed that more than 50% of doctoral students drop out of their programs, with studies showing that factors such as stress, anxiety, exhaustion, and disinterest contribute to their decisions.^14^

It can be difficult to understand the mental health status of the graduate population as unique from undergraduate students as most research studies combine both the samples.^15^ However, we do know that graduate students face several unique challenges due to changes in demographics and social patterns. There are generational factors as well as today’s graduate students are more likely to have several familial and financial responsibilities as they enter graduate school compared to preceding generations. Unlike undergraduates, graduate students usually work in an atmosphere that provides less direction, requiring them to have considerable self-motivation in organizing their progress through graduate programs.^16^

Despite the fact that the overall mental health of Canadian graduate students has become a priority concern in many institutions,^17^ there is limited research on this subject that looks particularly at graduate students. Understanding the breadth of literature in this area will provide a foundation for universities in Canada to refine, design, and implement meaningful mental health services for graduate students. Mental health issues not only affect the personal well-being of graduate students but also impede their academic success, professional growth, and contributions to society. Addressing this issue through informed interventions is essential for improving the quality of graduate education and equipping students to thrive in their academic and professional journeys.

As far as we are aware, there has not been any systematic or scoping reviews conducted to synthesize literature on the mental health of graduate students in Canada. This lack of consolidated research creates barriers to understanding the specific needs of this population and developing targeted mental health programs. The findings will not only shed light on the state of graduate student mental health in Canada but also provide actionable insights for stakeholders to prioritize mental health initiatives tailored to this vulnerable population. Ultimately, this study contributes to the growing recognition of the mental health crisis among graduate students and offers a foundation for evidence-based policy and program development at Canadian universities.

To address this gap in literature, we conducted a scoping review guided by the research question: *What is the scope and nature of peer-reviewed empirical literature published on graduate student mental well-being in Canada?*

## Method

We conducted a scoping review following the methodological framework by Arksey and O'Malley,^18^ using PRISMA-ScR guidelines.^19^ (See appendix 1 for completed Prisma checklist). The framework consists of 5 steps: identifying the research question, identifying relevant studies, selecting appropriate studies, charting the data, and collating, summarizing, and reporting the results. Due to resource limitations, we did not conduct a formal consultation step. However, we engaged in thorough discussions among the co-authors, who are experts in the field of mental health. These discussions allowed us to integrate diverse perspectives and insights into the review process, ensuring a more comprehensive analysis of the existing literature on the mental well-being of graduate students in Canada. A description of each of these processes is presented below. Our review was registered prospectively with the Open Science Framework in May 2023.

### Search Strategy

We collaborated with an academic research librarian to develop a comprehensive search strategy covering 7 databases: EMBASE, CINAHL, PsycInfo, Medline, Sociological Abstracts, Nursing and Allied Health, and ERIC. Our search encompassed data available until April 2023. Using Boolean “AND”, we combined mental health, graduate student, and Canada as search concepts, adapting terms for each database. A sample EMBASE search is detailed in Appendix 2. Additionally, we examined reference lists for supplementary articles. Inclusion criteria encompassed studies focusing on graduate students’ mental well-being in Canada, published in English, and employing empirical designs. Non-peer-reviewed articles, study protocols, dissertations and theses, and conference abstracts were excluded.

#### Study Selection

We used Covidence™, a collaborative review software,^20^ to upload and manage our database searches. Two raters (EJ, RB) independently assessed titles and abstracts based on inclusion and exclusion criteria. Full texts of relevant papers were then reviewed by the same raters to determine eligibility. Disagreements were resolved through consensus or consultation with a third rater (CM).

#### Data Extraction

We utilized a custom data extraction form developed in Covidence,^20^ to extract information from included studies: author(s); year of publication; study design; methodology; clinical characteristics of participants; number of participants; demographic characteristics of participants, journal discipline and university.

#### Narrative Synthesis

We used Dedoose,^21^ a qualitative data management program, to perform qualitative content analysis. This analysis helped us identify key information from the included studies and synthesize it in a precise and rigorous manner. We coded relevant statements from the studies, conducted inductive coding, and organized the codes into categories and subcategories to develop themes and sub-themes. This analysis allowed us to create a narrative summarizing the current research and identify shared aspects of the work.

### Findings

**Figure 1.**
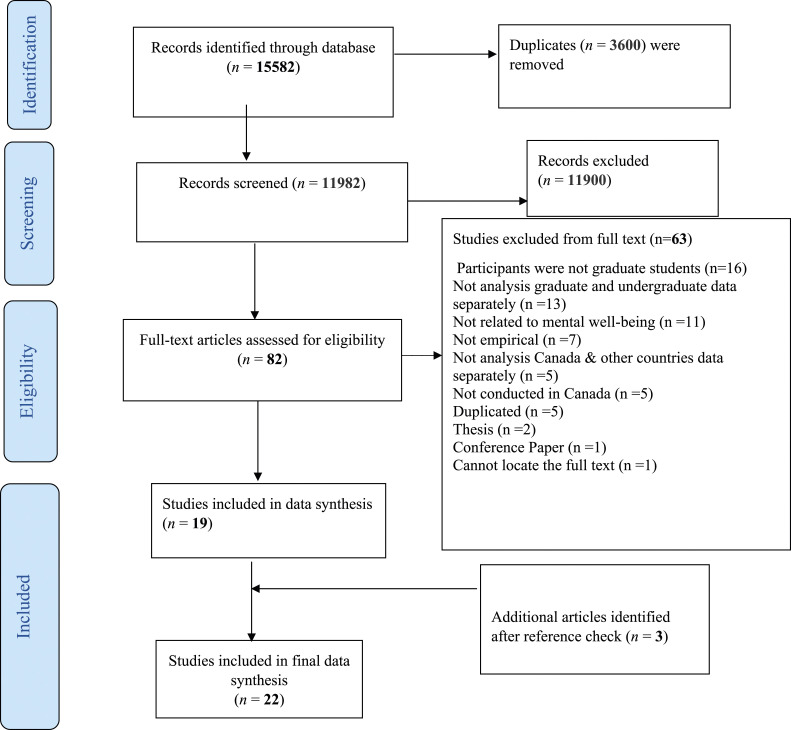
Prisma Diagram.

Our initial search yielded 15,582 citations, reduced to 11,982 after removing duplicates. Following title and abstract screening, 11,900 articles were excluded, leaving 82 for full-text review. Three additional articles were found through reference list searches. Ultimately, 22 articles met inclusion criteria. Figure 1 displays the PRISMA flow diagram detailing this process, including reasons for exclusion.

This review analyzed 22 articles on graduate students’ mental health in Canada. The studies used a combination of quantitative, qualitative, and mixed methods. Participants were from various universities across Canada, including the University of Calgary (4), University of Western Ontario (4), York University and University of Toronto (3), University of Toronto (1). University of Manitoba (2), University of Alberta (2), University of British Columbia (1), McGill University (1), Wilfrid Laurier University (1), and Ontario Universities as a whole (1). Lastly, there were 2 (2) study that included participants from various locations across Canada. The articles were published in psychology, health science, interdisciplinary, education, nursing, psychiatry, medicine, and women’s studies journals. The studies ranged from 1966 to 2023. For more detailed information, please refer to Table 1 for individual study characteristics and Table 2 for publication characteristics.

**Table 1.** Description of Included Studies (n = 22).

Authors	Study design	Methodology*	Program students were studying	Clinical characteristics of the sample???	Number of participants	Demographic characteristics of the sample	Journal discipline	University
Acker and Haque (2015)	Qualitative	Semi-structured interview	Unspecified	Unspecified	27	Age: Unspecified	Education	University of Toronto
						Gender: n = 5 men; n = 22women		
						Race/Ethnicity: Black :10 White: 4 Other: 13 LGBTQ2: Unspecified		
Baghoori et al (2022)	Quantitative	Cross-sectional study	Engineering (110) Science (91) Arts (43) Medicine and Dentistry (23) Law and Business (17) Rehabilitation and Kinesiology (15) Nursing (8) Education and native studies (6)	Unspecified	338	Age: 18-22: N = 145 23-28: N = 120 +29: N = 73 Gender: n = 129 men; n = 203 women; n = 6 prefer not to say and other Race/Ethnicity: 240 international students were from Asian countries, 35 from South American countries, 27 from African countries, 20 from North American countries, 14 from European countries, and 1 from New Zealand LGBTQ2: Unspecified	Health science	University of Alberta
Beks et al (2018)	Quantitative	Survey	Unspecified	Unspecified	354	Age: 17-40 Gender: Male (33.1%) Female (64.4%) Race/Ethnicity: Participants were predominately Caucasian LGBTQ2: Transgender (1.1%) Gender Fluid (1.4%)	Education	University of Calgary
Boyce and Barnes (1966)	Quantitative	Survey	Unspecified	The diagnoses in the groups were quite varied, consisting of “depressive reaction”, “anxiety reaction”, personality disorder, “adjustment reaction”, and schizophrenia, in that order of frequency. The problem of sexual identity was obvious in at least 6 of the cases	97	Unspecified	Psychiatry	University of Western Ontario
Clarke (2023)	Quantitative	Survey	Unspecified	Unspecified	6685	See Table 2. Page 286	Interdisciplinary	Wilfrid Laurier University
Cowie et al (2018)	Quantitative	Survey	Social sciences (43.1%), sciences (33.1%), Health professions (7.4%)	Unspecified	269	Age: 30.7 Gender: n = 128 men; n = 141 women Race/Ethnicity: Caucasian (59.5%), Asian (15.2%), other ethnicities (12.6%), or not disclosed (12.6%) LGBTQ2: Unspecified	Interdisciplinary	University of British Columbia
Didehvar and Wada (2023)	Qualitative	Phenomenology	Engineering, science, and social sciences	Unspecified	6	Age: Unspecified Gender: n = 2 men; n = 4 women Race/Ethnicity: All participants were Iranian LGBTQ2: Unspecified	Psychology	University of Calgary
Fried et al (2019)	Mixed method	Experimental	Education, Health sciences, Information and Media studies, Affiliate University College Medicine and Dentistry (MSc/PhD, Not MDs), science, social sciences	Unspecified	11	Age: 22-31 Gender: n = 3 men; n = 7women Race/Ethnicity Caucasian (4);East Asian (2); Hispanic (1); Middle Eastern (3); South Asian (1) LGBTQ2: Genderqueer female	Psychology	University of Western Ontario
Fried et al (2022)	Mixed method	Semi-structured interview and survey	See Table 2. Page 902	Unspecified	NA	See Table 2. Page 902	Health science	University of Western Ontario
Helmers et al (1997)	Quantitative	Survey	Unspecified	Unspecified	215	Unspecified	Health science	McGill University
Laurencelle and Scanlan (2018)	Qualitative	Phenomenological approach	Nursing	Unspecified	15	Age = 36-60 Gender: All women Race/Ethnicity: Unspecified LGBTQ2: Unspecified	Nursing	University of Manitoba
Longfield et al (2006)	Qualitative	Focus group interview	Occupational therapy (22) Other disciplines (25)	Unspecified	47	Unspecified	Interdisciplinary	University of Western Ontario
McEwan and Goldenberg (1999)	Quantitative	Descriptive correlational design	Nursing	Unspecified	39	Age: 20-29: N = 9; 30-39: N = 12; 40-49; N = 18 Gender: n = 0 men; n = 39 women Race/Ethnicity: LGBTQ2: Unspecified	Nursing	Three large Ontario universities
Mongrain and Blackburn (2005)	Quantitative	Retrospective and prospective design	Unspecified	Unspecified??	97	Age: mean: 28 Gender: 79% female, 21% male Race/Ethnicity: Caucasian (81%), Asian (7%), Other (7%), Hispanic (1%) and Black (1%), while 1% did not say LGBTQ2: Unspecified	Interdisciplinary	York University and University of Toronto
Peluso et al (2011)	Quantitative	Survey	Psychology	Unspecified	292	Age: Unspecified Gender: 87% Race/Ethnicity: LGBTQ2: Unspecified	Psychology	From across Canada
Peynenburg et al (2020)	Quantitative	Survey	Unspecified	See Table 1. Page 222	Undergraduate: 236 Graduate: 78	Age: 27.4 Gender: Male (49.7) Female (50%); prefer not to answer (0.3%) Race/Ethnicity: Asian (eg, Chinese, Japanese) (19%) Hispanic (3.8) First Nations/Metis (4.1%) African (5.1%) Middle Eastern (3.2%) Caucasian/European (51.7%) South Asian (7%) Other (5.7%) LGBTQ2: Unspecified	Psychology	From across Canada
Schroeder et al (2019)	Quantitative	Survey	Psychology program & Counselling program	Unspecified	78	Age = 19-24: (11); 25-29: (31); 40-49 (18) Gender: Women: 75 Men: 3 Race/Ethnicity: Unspecified LGBTQ2: Unspecified	Education	University of Calgary
Sturman et al (2006)	Quantitative	Survey	Unspecified	Participants had at least 1 prior episode of major depression	102	Age = Unspecified Gender: Woman (77); Men (25) Race/Ethnicity: Unspecified LGBTQ2: Unspecified	Psychology	University of Toronto and York University
Sturman & Mongrain. (2005)	Quantitative	Cross-sectional	Unspecified	Participants had at least 1 prior episode of major depression	146	Age = 23-54 Gender: Woman (105); Men (41) Race/Ethnicity: The majority of the sample was Caucasian (121), followed by Asian (5), Hispanic (4), Black (4), and other heritage 10) LGBTQ2: Unspecified	Psychology	University of Toronto and York University
Toews et al (1993)	Quantitative	Survey	Medical students: 216 Residents: 279 MSc/PhD students: 167	Unspecified	662	Age: mean: Medical students:26.4 Residents: 30.7 MSc/PhD students: 29.93 Gender: n = 231 men; n = 173women Race/Ethnicity: Unspecified LGBTQ2: Unspecified	Medicine	University of Calgary
Wall (2008)	Qualitative	Exploratory??	Arts-related disciplines	Unspecified	9	Age = mid-20s to late-40s Gender: all participants were women Race/Ethnicity: Unspecified LGBTQ2: Unspecified	Women’s studies	University of Alberta
Webber et al (2022)	Mixed method	Questionnaires and inductive analysis of focus group	Occupational therapy & physical therapy	Unspecified	98	Age: mean: 28 Gender: 75 females, 23 male Race/Ethnicity: Unspecified LGBTQ2: Unspecified	Health science	University of Manitoba

Terminology/descriptions are based on information provided in the papers.

**Table 2.** Publication Characteristics (n = 22).

Study Characteristics	n (%)
Study design	
Quantitative	14 (63.6)
Cross-sectional	
Qualitative	5 (22.7)
General qualitative	
Mixed methods	3 (13.5)
Discipline	
Psychology	6 (27.2)
Interdisciplinary	4 (18.1)
Health science	4 (18.1)
Education	3 (13.6)
Nursing	2 (9)
Psychiatry	1 (4.5)
Medicine	1 (4.5)
Women’s studies	1 (4.5)
University	
University of Calgary	4 (18.1)
University of Western Ontario	4 (18.1)
York University and University of Toronto	3 (13.6)
University of Manitoba	2 (9)
University of Alberta	2 (9)
University of Toronto	1 (4.5)
University of British Columbia	1 (4.5)
McGill University	1 (4.5)
Wilfrid Laurier University	1 (4.5)
Ontario Universities	1 (4.5)
From Across Canada	2 (9)

Note: Percentage sums do not all equal 100 due to rounding.

### Narrative Synthesis

We generated 3 main themes during our analysis of the content: (1) Determinants of mental health in graduate students are myriad; (2) Coping Strategies for Graduate Student Stress; and (3) Bridging Support: Enhancing Mental Well-Being. In Table 3, the articles that are related to these themes are listed and described.

**Table 3.** Themes Represented by Included Studies.

Theme	Studies included
The Determinants of Graduate student Mental Health are Myriad (n = 17)	12, 17, 22, 23, 24, 25, 26, 27, 28, 29, 30, 31, 32, 33, 34, 35, 36
Coping strategies for Graduate student stress (n = 9)	17,24,29,36,37,38,39,40,41
Bridging support: Enhancing Mental Well-Being (n = 3)	12,29,24

### The Determinants of Graduate Student Mental Health are Myriad

The determinants of mental health in graduate students were explored in 17 studies. (See Table 3). The authors of 11 of included studies discussed how sociodemographic factors impact the mental health in graduate students.^12,17,22-30^ In McEwan & Goldenberg’s study, unemployed graduate students had higher anxiety levels compared to employed students. Older students aged 30-39 had lower anxiety levels than younger students. Cowie^23^ found that women experienced more academic stress than male students. Peluso’s^12^ study discussed mental health issues in psychology graduate students, with 33% reporting clinically significant symptoms of depression. They recommended identifying specific variables to help mitigate the negative effects of depression in these programs.

The impact of level of study was also explored in 7 studies.^17,24-29^ The participants in Fried’s study^24^ believed that their sources of stress had changed from their time as undergraduate students, as they now have additional responsibilities in addition to school work. Webber^17^ also reported that occupational and physical therapy graduate students had higher levels of stress, anxiety and/ or depression compared to undergraduate students. According to students in this study, their level of control over their schedule decreased in graduate school compared to their time as undergraduates. They found themselves spending a significant amount of time in classes, which left them with limited free time. Additionally, students expressed instructors had high expectations for them in graduate school, causing some uncertainty about their academic requirements. Boyce^25^ also found that the number of graduate students seeking help at the Department of Psychiatry, Victoria Hospital (London, Ontario), was twice as high compared to the overall students population at the university.

Graduate students exhibited distinct attitudes toward certain treatments compared to undergraduates. For instance, in Peynenburg’s study,^26^ they showed a more favorable view of internet-delivered cognitive behavior therapy (ICBT) and considered it more credible. Fried^24^ noted that undergraduates may feel isolated due to busy schedules and housing issues, while graduate students may experience isolation from independent thesis work. Interestingly, graduate students preferred accessing mental health support separate from undergraduates, perhaps to avoid encountering their own students while seeking help. Baghoori’s^27^ research at the University of Alberta found significant variations in approach coping among undergraduate, master’s, and Ph.D. students. Ph.D. students, particularly, demonstrated more frequent use of coping techniques, reflecting their experience in handling academic stress. This indicates their adeptness in maintaining a healthy work-life balance and effectively addressing challenges during their program, relying on practical coping strategies to find solutions.

In Fried’s^24^ study, graduate students reported increased resilience compared to their time as undergraduates, attributing it to overcoming challenges. However, Beks’^28^ study suggests that both undergraduate and graduate students have similar levels of resilience and mental health literacy. In Longfield’s^29^ study, students mentioned a shift in their self-worth as they transitioned to graduate studies, focusing more on their own sense of worth rather than seeking validation externally.

Didehvar & Wada’s study^30^ argued that international graduate students may have totally different challenges as domestics; potentially leading to more or different sources of stress impacting their mental health and wellbeing the mental health including feelings of isolation and loneliness resulting from cultural adaptation. Addressing existential worries from acculturation stress is crucial, recommending environments fostering authentic conversations about these concerns. Clarke’s^31^ research at Wilfrid Laurier University compared mental health between international and domestic graduate students, noting that visits to psychiatry may not accurately reflect mental health and wellbeing status of both international and domestic graduate students. Both groups faced similar stressors like depression and finances, yet more international students experienced homesickness and roommate difficulties. Stress and anxiety were common hindrances to academic performance. International students sought mental health services more than domestic students, possibly influenced by cultural perspectives and mental health literacy disparities. Additionally, fewer international students expressed intent to seek future mental health support, potentially influenced by cultural factors and differing levels of mental health awareness.

Sturman & Mongrain’s study^32^ examined 146 graduate students with a history of major depressive episodes, revealing that self-critical individuals often feel trapped within their thoughts and perceive themselves negatively compared to others, irrespective of mood. Mongrain & Blackburn^33^ found that sociotropy, perfectionism, and negative inferential styles about academic failure were linked to chronic depression among graduate students. Cowie^23^ highlighted neuroticism’s role in vulnerability to distress disorders and its association with negative thought patterns, while perfectionism correlated with academic issues like stress and imposter syndrome. Sturman et al^34^ discovered that attributional style predicts hopelessness depression in graduate students. McEwan & Goldenberg’s^22^ research indicated trait anxiety’s positive correlation with academic success and negative correlation with state anxiety, suggesting effective coping mechanisms despite high state anxiety levels. These findings collectively underscore the complex interplay between psychological factors and academic performance among graduate students.

Two studies^12,35^ found that insufficient funding negatively impacts the mental health of graduate students. In Acker’s study,^35^ students expressed anxiety due to inadequate financial support and the struggle to secure assistantships and scholarships. The importance of supervisor/academic advisory relationships was discussed in 2 studies. In Peluso’s study,^12^ experimental psychology students linked their mental well-being to satisfaction with these relationships. Fried’s study^24^ revealed stressors related to impressing supervisors/superiors, with participants noting outdated beliefs and negative behaviors perpetuated by faculty members.

Fried’s study^36^ emphasized physical activity’s importance for graduate students’ mental health, despite challenges like costly gym classes. Balancing academics, exercise, and sleep was difficult, with varying success. Participants desired integrated recreational and mental health services. At the University of Western Ontario, Fried and colleagues^36^ found that the Breaking Grad program’s peer mentorship improved mental health, fostering self-awareness, skills, diverse perspectives, and a supportive community.

### Coping Strategies for Graduate Student Stress

Participants in 9 studies described their feeling of stress during graduate education.^17,24,29,36-41^ In Fried’s study,^36^ all participants shared their experiences of ongoing stress and anxiety. Laurencelle & Scanlan’s^39^ study highlighted graduate students’ stress stemming from academic demands like grant deadlines and unexpected research challenges. Participants shared experiences of stress impacting academic performance, emphasizing the challenges inherent in graduate studies. They also discussed how these moments of stress affected their academic achievement. Participants in Fried’s study^24^ specifically mentioned feeling isolated as graduate students, which made it challenging for them to make friends or find a support network. Additionally, they shared that the competitive nature of graduate school created a negative and stressful atmosphere where comparing themselves to others was the norm. In Webber’s study^17^ also some students experienced feelings of inadequacy and stress when they became aware that their peers comprehended the materials they struggled with, or when their classmates completed assignments ahead of them. However, some students expressed that being surrounded by other students brought them a sense of support, and they experienced a sense of stress when they found themselves alone at the end of the day. In 5 of included studies^17,24,36,39,41^ graduate students were concerned about their work-life balance. One of the female participants in Wall’s study^41^ expressed that “*we’re not allowed to be real human beings in academia. It’s like you’re not supposed to have a life extraneous to academia.”* Another participant in this study had significant distress about having to delay children due to pursuing a doctoral study and it led her to choose to quit her program. Occupational and physical therapy graduate students in Webber’s study^17^ discussed the irony of their situation. Despite being in professions that prioritize health and balanced lifestyle, they found that their learning environment did not support this. The students expressed their disappointment and attributed the irony to the lack of time for physical activity or the challenges they faced in incorporating it into their routines. Participants in Fried’s study^36^ also expressed worries about heavy workloads often resulted in them to prioritizing their studies above health. Participants in 4 studies also discussed how they cope with school-related stress.^17,29,36,40^

In Fried’s study,^36^ graduate students engaged in a peer coaching program to tackle stress, gaining diverse perspectives that improved stress management. Similarly, Longfield found that social and physical activities alleviated stress. Webber’s^17^ research highlighted the dilemma of balancing personal life and academics, with social media aiding stress coping for some but exacerbating it for others. Some students redirected focus to controllable aspects to manage stress. Coping methods varied, emphasizing individualized approaches. Schroeder’s^40^ study proposed deadline flexibility, like a late submission option, as a stress-reduction measure. These findings collectively underscore the multifaceted nature of stress management among graduate students, suggesting diverse strategies and interventions tailored to individual needs. Peer support programs, engagement in recreational activities, mindful attention to controllable factors, and flexible assignment deadlines emerge as potential avenues for mitigating stress in academia. Such insights contribute to fostering healthier and more supportive academic environments conducive to graduate student well-being.

### Bridging Support: Enhancing Mental Well-Being

In 3 of the included studies, the importance of support systems to improve the mental well-being of graduate students was discussed.^12,24,29^ In Fried’s study^24^ all students acknowledged that the host institution offers a wide range of services; however, they expressed that accessing these services was challenging. All participants shared their concerns about the difficulty they faced in finding the right kind of support when they did not necessarily need to see a counselor, but still needed some form of assistance. Specifically, they expressed a desire for peer programming in such cases. Some students were placed on long waitlists of up to 4 months to access services. Sometimes, they are only allowed to have 1 counseling session or were turned away if they were not considered to be in an immediate crisis. Students in Fried’s^24^ study also mentioned that receiving counseling services outside of campus can be quite expensive. Graduate students often have different needs compared to undergraduate students, so they may prefer to seek help from counselors who are specialized in dealing with graduate students and who are not affiliated with the undergraduate students they teach.^24^ In Peluso’s study^12^ the importance of providing mental health support systems for clinical and counseling students was discussed. These individuals might feel hesitant seek help within their communities due to their training environment and clinical placements. It emphasizes the need for promoting openness and help-seeking behavior, particularly among clinical and counseling students.

## Discussion

In this scoping review, we have identified 22 empirical studies exploring mental well-being of graduate students in Canada. The purpose of this review was to summarize and analyze all existing literature in order to provide guidance for future research, policy and practice in Canada. A preliminary analysis indicates that while awareness of mental health among graduate students has increased over the decades, significant gaps remain in addressing the unique challenges faced by this demographic. Early studies primarily focused on general stressors, while more recent research highlights specific issues such as the impact of financial stress and the importance of supportive academic environments. By examining literature dating from 1966 to 2023, we can conclude that the evolving understanding of mental health issues underscores a growing recognition of the multifaceted nature of graduate student well-being, suggesting that continued research is essential for developing targeted interventions**.** We identified 3 themes in our narrative synthesis that highlight the importance of focusing on the mental well-being of graduate students in Canadian universities: (1) Determinants of mental health in graduate students are myriad; (2) Coping Strategies for Graduate Student Stress; and (3) Bridging Support: Enhancing Mental Well-Being.

The findings of our narrative synthesis indicate that there are several determinants that influence the mental health of graduate students. The sociodemographic determinants include employment status, age, gender, program type, level of studies, and being an international graduate student. In a study conducted by McEwan and Goldenberg,^22^ it was observed that graduate school participants who were not working had a greater level of anxiety compared to those who were employed. Additionally, the study found that older students, specifically those between 30 and 39 years old, experienced lower levels of anxiety when compared to younger students. The sociodemographic variables that predict psychological well-being in international students was explored in a study conducted in Germany by Akhtar & Kroener-Herwig.^42^ According to this study, among variables such as age, gender, marital status, subject of the study, degree, and major source of financial support, gender was the only predictor reaching significant values. In this study male students displayed a higher level of psychological well-being when compared to their female peers. This result is consistent with the findings of 1 of our included studies conducted in Canada that showed female graduate students were found to be more prone to experiencing academic stress in comparison to male graduate students.^23^

Results from this scoping review also revealed the mental health differences between undergraduate and graduate students. The results showed that graduate students and undergraduate students in Canada are different in source and level of stress,^17,24^ attitude toward treatment,^26^ reasons for experiencing isolation,^24^ utilizing coping strategies,^27^ sense of resilience^24,28^ and self-worth.^29^ The mental health differences between graduate and undergraduate students are also discussed in literature. In a study conducted by Wyatt & Oswalt^43^ in the United States, even though graduate students reported fewer mental health concerns compared to undergraduates, they did acknowledge experiencing more stress due to schoolwork, finances, graduate/teaching assistantships, career planning, and family issues. The authors of this study believe that having a comprehensive understanding of the distinct mental health concerns experienced by undergraduate and graduate students is essential for providing effective assistance and support services to them. Despite increasing discussion of graduate students’ mental health in Canada, there has been a lack of emphasis on understanding the unique challenges faced by international students in this context. International graduate student mental health was discussed in only 2 of the included studies.^30,31^ While several studies have compared the prevalence of mental health conditions between international and non-international students in the literature, most of these studies include only undergraduate students^44^ or their sample has a small percentage of graduate students.^45^ As the number of graduate students in Canada continuing to increase,^46^ it is anticipated that there will also be an increase in the enrollment of international students in graduate studies. We believe that failing to address the unique needs of these international graduate students can have significant consequences, as dropout rates remain a major concern in graduate education. Therefore, it is crucial to identify effective strategies to provide support and assistance to international graduate students.

In 2 included studies,^12,35^ graduate students shared their thoughts on how their mental well-being is affected by a lack of adequate Results from this scoping review also revealed the mental health differences between undergraduate and graduate students. The results showed that graduate students and undergraduate students in Canada are different in source and level of stress,^17,24^ attitude toward treatment,^26^ reasons for experiencing isolation,^24^ utilizing coping strategies,^27^ sense of resilience^24,28^ and self-worth.^29^ The mental health differences between graduate and undergraduate students are also discussed in literature. In a study conducted by Wyatt & Oswalt^43^ in the United States, even though graduate students reported fewer mental health concerns compared to undergraduates, they did acknowledge experiencing more stress due to schoolwork, finances, graduate/teaching assistantships, career planning, and family issues. The authors of this study believe that having a comprehensive understanding of the distinct mental health concerns experienced by undergraduate and graduate students is essential for providing effective assistance and support services to them. Despite increasing discussion of graduate students’ mental health in Canada, there has been a lack of emphasis on understanding the unique challenges faced by international students in this context. International graduate student mental health was discussed in only 2 of the included studies.^30,31^ While several studies have compared the prevalence of mental health conditions between international and non-international students in the literature, most of these studies include only undergraduate students^44^ or their sample has a small percentage of graduate students.^45^ As the number of graduate students in Canada continuing to increase,^46^ it is anticipated that there will also be an increase in the enrollment of international students in graduate studies. We believe that failing to address the unique needs of these international graduate students can have significant consequences, as dropout rates remain a major concern in graduate education. Therefore, it is crucial to identify effective strategies to provide support and assistance to international graduate students.

Several studies have been conducted worldwide regarding the impact of financial problems on the mental health of graduate students.^47-50^ Hyun^47^ conducted quantitative study on mental health of international graduate students in United States found that higher financial confidence was significantly associated with lower use of mental health services. The finding of this study also showed that international students who had a functional relationship with their advisors were less likely to experience emotional or stress-related issues in the past year. This finding is consistent with 2 of included studies in this review,^12,24^ in which, the level of satisfaction that students experienced in their supervisory or academic advisory relationship played a significant role in determining their overall mental well-being. We believe that the impact of financial issues and functional relationship with supervisors/advisors on mental health of graduate students should be explored more in the context of Canadian universities. Collaborative efforts between graduate program administrators, funding agencies, and other stakeholders are essential in ensuring that graduate students have sufficient funding throughout their studies.

One of the included studies^36^ found that physical activity positively affects the mental well-being of graduate students, which aligns with existing literature.^51-53^ While there is limited research specifically examining the impact of physical activity on mental health within Canadian graduate student populations, numerous studies in broader contexts have consistently shown that physical activity is associated with improved mental health outcomes across various demographics.^54-56^ This suggests that the benefits of physical activity observed in other populations are likely applicable to Canadian graduate students as well. Therefore, promoting physical activity within this demographic could be a vital strategy for enhancing their overall mental health and well-being.

### Implications for Policy and Practice

This review underscores the sparse research on Canadian graduate students’ mental health. While 63.6% of the studies in this review employed quantitative methods, qualitative research offers a deep insight into students' experiences. Conducting more qualitative research is recommended to achieve a comprehensive understanding. Coordinated nationwide qualitative studies can bridge the research-practice gap, ensuring tailored support systems. Open dialogue and consideration of students’ stories can foster a supportive campus environment. Disseminating findings widely can raise awareness and destigmatize mental health issues. It’s crucial to involve diverse graduate students in policymaking and provide training for advisors and faculty to better support them.

### Limitations

Our findings contribute to the literature, but there are notable limitations. Firstly, as a scoping review, we did not assess varying study qualities. Secondly, we solely focused on peer-reviewed journal studies, potentially missing insights from grey literature like government papers. Lastly, due to resource constraints, we did not involve stakeholders in a consultation step, which could enhance knowledge exchange.^57^

## Conclusion

This study highlights the multifaceted nature of mental well-being among graduate students in Canada, revealing 3 key themes: the myriad determinants of mental health among graduate students, effective coping strategies for managing stress, and the need for bridging support to enhance overall mental well-being in Canadian universities. By recognizing these themes, we can better understand the unique challenges faced by this demographic. It is crucial for universities to create supportive environments that address these challenges, ensuring that graduate students receive the assistance they need to thrive academically and personally.

## So What? Implications for Health Promotion Practitioners and Researchers

In light of the findings from this scoping review, prioritizing research on the mental health of graduate students in Canada is essential. Future studies should focus on the unique experiences of diverse demographics, including international students, underrepresented groups, and those with disabilities, to uncover the specific challenges they face, such as the effects of discrimination, work-life balance, and varying levels of social support. Additionally, researchers should explore the role of academic environments and institutional policies in shaping mental health outcomes, emphasizing the importance of supportive mentorship, accessible mental health services, and inclusive campus cultures.

Incorporating longitudinal studies will provide insights into the long-term impacts of graduate education on mental health, while qualitative research methods, such as in-depth interviews and focus groups, could capture the nuanced experiences and coping strategies of these groups. Moreover, it would be beneficial to investigate the effectiveness of specific interventions, such as peer mentorship programs and financial support initiatives, on improving mental well-being. By focusing on these areas, future research can guide the development of tailored interventions and support mechanisms within Canadian universities, ensuring that all graduate students have the resources they need to thrive both academically and personally.

## Supplemental Material

Supplemental Material - The Mental Well-Being of Graduate Students in Canada: A Scoping Review Supplemental Material for The Mental Well-Being of Graduate Students in Canada: A Scoping Review by Elham Javadizadeh, Abram Oudshoorn, Lori Letts, Skye Barbic, and Carrie Anne Marshall in American Journal of Health Promotion

Supplemental Material - The Mental Well-Being of Graduate Students in Canada: A Scoping Review Supplemental Material for The Mental Well-Being of Graduate Students in Canada: A Scoping Review by Elham Javadizadeh, Abram Oudshoorn, Lori Letts, Skye Barbic, and Carrie Anne Marshall in American Journal of Health Promotion
