# Supplementary material for: The Mental Well-Being of Graduate Students in Canada: A Scoping Review
Source: Am J Health Promot. 2025 Mar 12;39(6):921–35. doi: 10.1177/08901171251326308 (PMC12144327; doi:10.1177/08901171251326308)
Supplement: Supplemental Material - The Mental Well-Being of Graduate Students in Canada: A Scoping Review [file sj-pdf-2-ahp-10.1177_08901171251326308.pdf]

Database: Embase Classic+Embase <1947 to 2023 April 21>

Search Strategy:

1 Mental well-being.mp. (5570)
2 Mental health.mp. (404950)
3 Mental disorder*.mp. (91594)
4 Psychosocial well-being.mp. (3086)
5 anxiety/ or Anxiety.mp. (500970)
6 Stress.mp. or physiological stress/ (1635543)
7 Acute stress disorder.mp. (2129)
8 Reactive attachment disorder.mp. (290)
9 Psycho*.mp. (1991298)
10 Psychotic disorders.mp. or psychosis/ (124232)
11 Schizophrenia*.mp. (250878)
12 Bi-polar.mp. (357)
13 Depression.mp. (868810)
14 Depress*.mp. (1007880)
15 Mood.mp. (178315)
16 Mental illness.mp. (50183)
17 Psychosocial.mp. (186833)
18 Personality disorder*.mp. (60023)
19 Conduct disorder*.mp. (11696)
20 Borderline disorder.mp. (169)
21 Obsessive-compulsive disorder*.mp. (36863)
22 OCD.mp. (17670)
23 Anorexia*.mp. (108688)
24 Bulimia*.mp. (17823)
25 Eating disorder*.mp. (48140)
26 Addict*.mp. (184090)
27 Alcoholism.mp. (156886)
28 Suicid*.mp. (166156)
29 Psychiatric disorder*.mp. (76501)
30 Mental disorder.mp. (16512)
31 Stigma*.mp. (77242)
32 Trauma*.mp. (675363)
33 PTSD.mp. or posttraumatic stress disorder/ (83675)
34 Graduate student*.mp. (7667)

35 Graduate.mp. (50898)

36 International student*.mp. (1260)

37 Master student*.mp. (138)

38 Grad*.mp. (1637285)

39 Advanced degree.mp. (692)

40 Doctorate.mp. (1636)

41 Doctoral.mp. (6938)

42 Postgrad.mp. (101)

43 Canada.mp. (283037)

44 Ontario.mp. (52654)

45 Alberta.mp. (16566)

46 Saskatchewan.mp. (4447)

47 Quebec.mp. (20778)

48 New Brunswick.mp. (1888)

49 Nova Scotia.mp. (4151)

50 British Columbia.mp. (15242)

51 Manitoba.mp. (5877)

52 Prince Edward Island.mp. (696)

53 Yukon.mp. (963)

54 Nunavut.mp. (739)

55 (Newfoundland and Labrador).mp. [mp=title, abstract, heading word, drug trade name, original title, device manufacturer, drug manufacturer, device trade name, keyword heading word, floating subheading word, candidate term word] (1329)

56 Northwest territories.mp. (767)

57 1 or 2 or 3 or 4 or 5 or 6 or 7 or 8 or 9 or 10 or 11 or 12 or 13 or 14 or 15 or 16 or 17 or 18 or 19 or 20 or 21 or 22 or 23 or 24 or 25 or 26 or 27 or 28 or 29 or 30 or 31 or 32 or 33 (5355970)

58 34 or 35 or 36 or 37 or 38 or 39 or 40 or 41 or 42 (1645437)

59 43 or 44 or 45 or 46 or 47 or 48 or 49 or 50 or 51 or 52 or 53 or 54 or 55 or 56 (327117)

60 57 and 58 and 59 (2838)
